# Crystal structure of Na_4_(As_2_O_5_)(H_2_O)_0.5_ and a survey of the pyroarsenite anion, (As_2_O_5_)^4−^

**DOI:** 10.1107/S2056989026002859

**Published:** 2026-03-24

**Authors:** Tobias Wolflehner, Matthias Weil

**Affiliations:** a TU Wien, Institute for Chemical Technologies and Analytics, Division of Applied Solid State Chemistry, Getreidemarkt 9/E164-05-1, 1060 Vienna, Austria; Universidad de la República, Uruguay

**Keywords:** crystal structure, diarsenite, hydrogen bonding, hemihydrate, hydro­flux synthesis, data mining

## Abstract

The title compound, tetra­sodium pyroarsenite hemihydrate, [Na_4_(As_2_O_5_)(H_2_O)_0.5_], represents the first pyroarsenite compound of an alkali metal. The asymmetric unit comprises four Na, two As, five O sites and one H site in a general position, and one water O atom located on a twofold rotation axis in space group *C*2/*c*. The (As_2_O_5_)^4–^ anion is made up from two trigonal-pyramidal {AsO_3_} units sharing a corner. In the crystal structure, all O atoms of the anion are also part of a framework defined by one [NaO_4_] and three [NaO_6_] coordination polyhedra. Almost linear hydrogen-bonding inter­actions of medium strength [O⋯O 2.651 (4) Å; O—H⋯O 171 (6)°] between the water mol­ecule and one of the terminal O atoms of the pyroarsenite anion consolidate the crystal structure. Data mining for structural data on isolated pyroarsenite anions resulted in 30 (As_2_O_5_)^4–^ groups present in various crystal structures. In these pyroarsenite anions, the mean As—O distance to terminal oxygen atoms is 1.764 (33) Å and to bridging oxygen atoms 1.856 (64) Å. The values of the As—O—As bridging angle are highly variable [range 107.78 (13) to 144.12 (5)°], with that of Na_4_(As_2_O_5_)(H_2_O)_0.5_ having by far the smallest value of all structures chacterized so far.

## Abstract 2

The crystal structure of Na_4_(As_2_O_5_)(H_2_O)_0.5_ consists of a half-eclipsed (As_2_O_5_)^4–^ anion embedded in a framework of [NaO_4_] and [NaO_6_] polyhedra. O—H⋯O hydrogen-bonding consolidates the packing.

## 1. Chemical context

Formation studies of alkali transition-metal oxido­anti­m­onates(V) and related oxidoarsenates(V) led to the unexpected discovery of the oxidoarsenate(III) compound K_3_FeAs_2_O_6_ under hydro­flux conditions (Wolflehner, 2026 ▸). A comprehensive review of this synthesis method has been given recently by He *et al.* (2023 ▸). To obtain the hypothetical sodium variant of K_3_FeAs_2_O_6_, the starting materials used under similar hydro­flux conditions have been adjusted. However, the corresponding experiment did not yield the planned target phase, but rather the transition metal-free compound Na_4_(As_2_O_5_)(H_2_O)_0.5_, the crystal structure of which is reported here, together with a survey on structural details regarding the pyroarsenite anion, (As_2_O_5_)^4–^.

## 2. Structural commentary

The asymmetric unit of Na_4_(As_2_O_5_)(H_2_O)_0.5_ comprises four Na, two As, six O and one H-atom positions. With the exception of the water O atom O1*W* (site symmetry 2; multiplicity 4, Wyckoff letter *e*), all atoms are located at sites corresponding to a general position (8 *f*) of space group *C*2/*c*.

The two As^III^ atoms each are coordinated by three oxygen atoms in a trigonal–pyramidal shape, typical for arsenite groups. Two such trigonal pyramids share an O atom (O3), resulting in the formation of the condensed (As_2_O_5_)^4–^ anion. The As—O bond lengths distribution in Na_4_(As_2_O_5_)(H_2_O)_0.5_ (Table 1 ▸) is typical for a condensed system consisting of two oxido anions, for example, for pyro-anions consisting of two tetra­hedral groups, *e.g.* phosphates (Durif, 1995 ▸) or silicates (Liebau, 1985 ▸), here with four shorter As—O bonds [average value 1.730 (5) Å] to terminal O atoms and two longer As—O bonds to the bridging O atom [average value 1.903 (12) Å]. The mean As—O bond length of 1.788 (82) Å in the anion of the title compound agrees very well with the literature value of 1.782 Å for the averaged As^III^—O distance in the crystal structures of arsenite compounds (Majzlan *et al.*, 2014 ▸). Structural data specific to pyroarsenite anions are discussed in more detail in section 3.

If the non-bonding 4*s*^2^ electron lone pair *ψ* of the As^III^ atom is also taken into account, [*ψ*AsO_3_] polyhedra with the shapes of flattened tetra­hedra are formed. The positions of *ψ* were calculated with the *LPLoc* program (Hamani *et al.*, 2020 ▸) resulting in the following fractional coordinates: *x* = 0.10919, *y* = −0.13221, *z* = 0.64501 for *ψ*1 located at the As1 atom [distance As1—*ψ*1 = 1.240 Å; radius(*ψ*1) = 1.19 Å], and *x* = 0.04042, *y* = 1.15526, *z* = 0.46720 for *ψ*2 located at the As2 atom [distance As2—*ψ*2 = 1.276 Å; radius(*ψ*2) = 1.19 Å].

**Figure 1:**
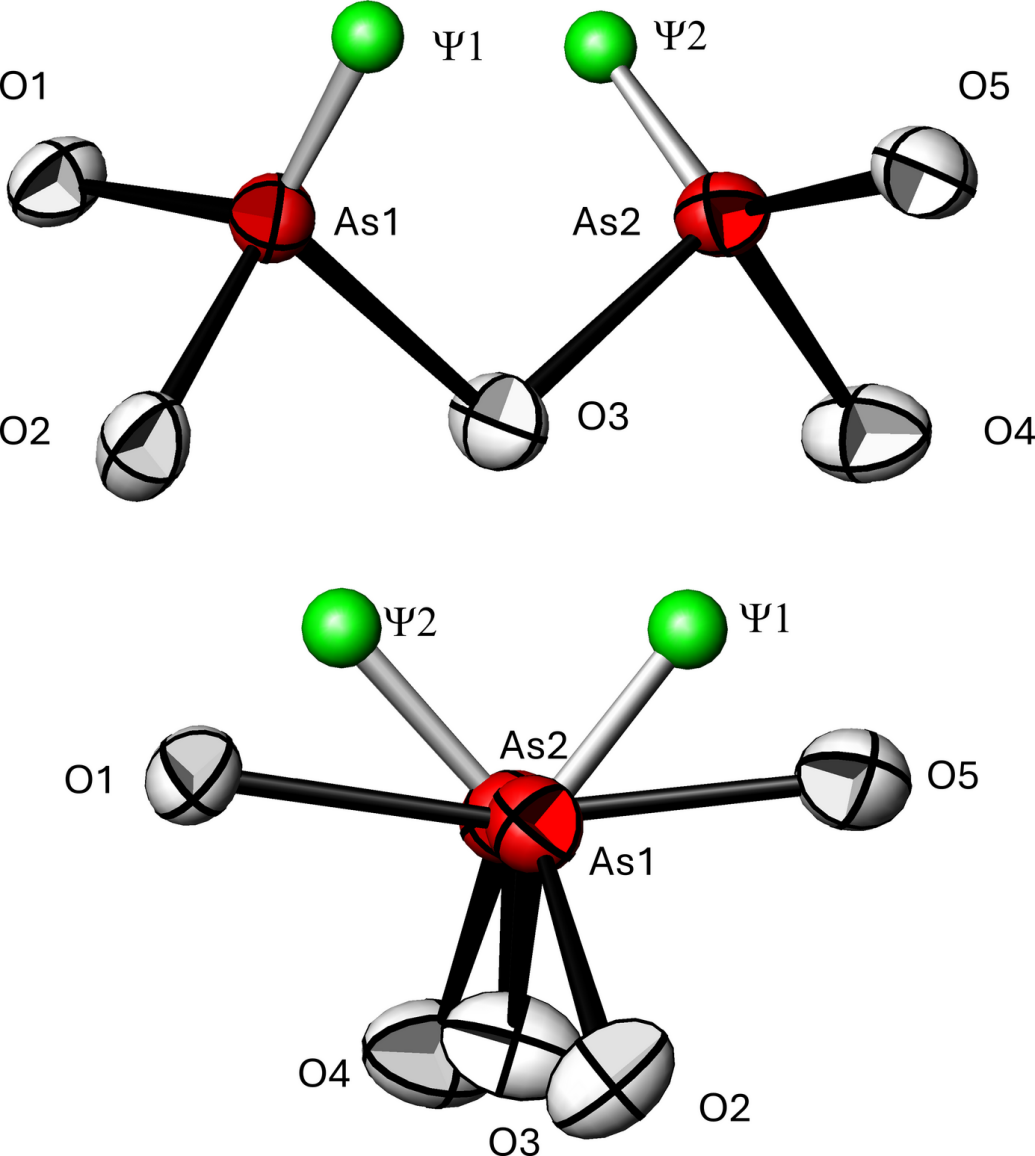
The (As_2_O_5_)^4–^ anion in the title compound in a side view (top) and approximately along the As1⋯As2 axis (bottom). Displacement ellipsoids are drawn at the 90% probability level; the calculated electron lone pairs *ψ* are indicated as spheres of arbitrary radius.

The pyroarsenite anion exhibits a half-eclipsed conformation as evidenced by the torsion angles O1—As1⋯As2—O4 of 82.71 (19)° (synclinal), O2—As1⋯As2—O5 of 81.15 (18)° (synclinal), O2—As1⋯As2—O4 of −30.5 (2)° (boundary between synperiplanar and synclinal) and O1—As1⋯As2—O5 of −165.61 (13)° (anti­periplanar). The free electron pairs (taking into account the coordinates given above) have a torsion angle *ψ*1—As2⋯As2—*ψ*2 of −80.34° and are therefore also synclinal to each other (Fig. 1 ▸).

**Figure 2:**
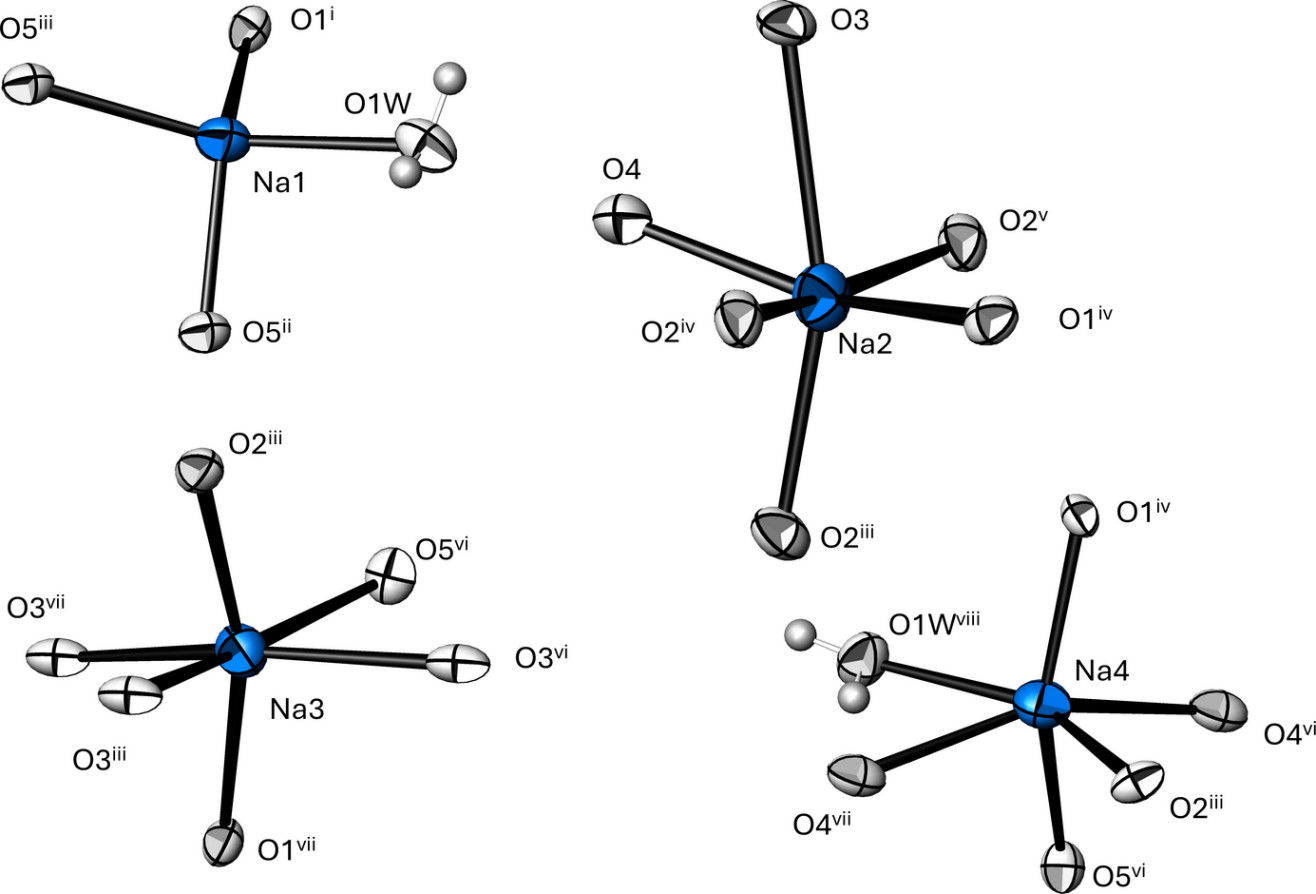
Coordination environments for the four Na^+^ positions. Displacement ellipsoids are as in Fig. 1 ▸. [Symmetry codes: (i) *x*, −*y* + 1, *z* − 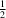; (ii) −*x*, *y* + 1, −*z* + 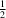; (iii) *x*, −*y*, *z* − 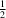; (iv) −*x* + 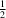, −*y* + 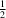, −*z* + 1; (v) −*x* + 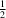, −*y* − 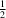, −*z* + 1; (vi) −*x* + 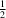, *y* + 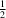, −*z* + 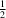; (vii) −*x* + 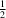, *y* − 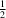, −*z* + 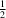; (viii) *x* + 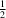, *y* − 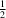, *z*.]

The Na^+^ sites show coordination numbers of 4 (Na1) and 6 (Na2, Na3 and Na4), and their coordination polyhedra are shown in Fig. 2 ▸. The description of the closest matching ideal polyhedron for each site and qu­anti­fication of the distortion (*δ*) from it is based on the *Polynator* program (Link & Niewa, 2023 ▸), and numerical data considering Na—O distances up to 3.0 Å as relevant are compiled in Table 2 ▸, including averaged Na—O bond lengths. The latter are in reasonable agreement with literature values (Gagné & Hawthorne, 2016 ▸) of 2.359 (76) Å for coordination number 4, and 2.441 (112) Å for coordination number 6.

**Figure 3:**
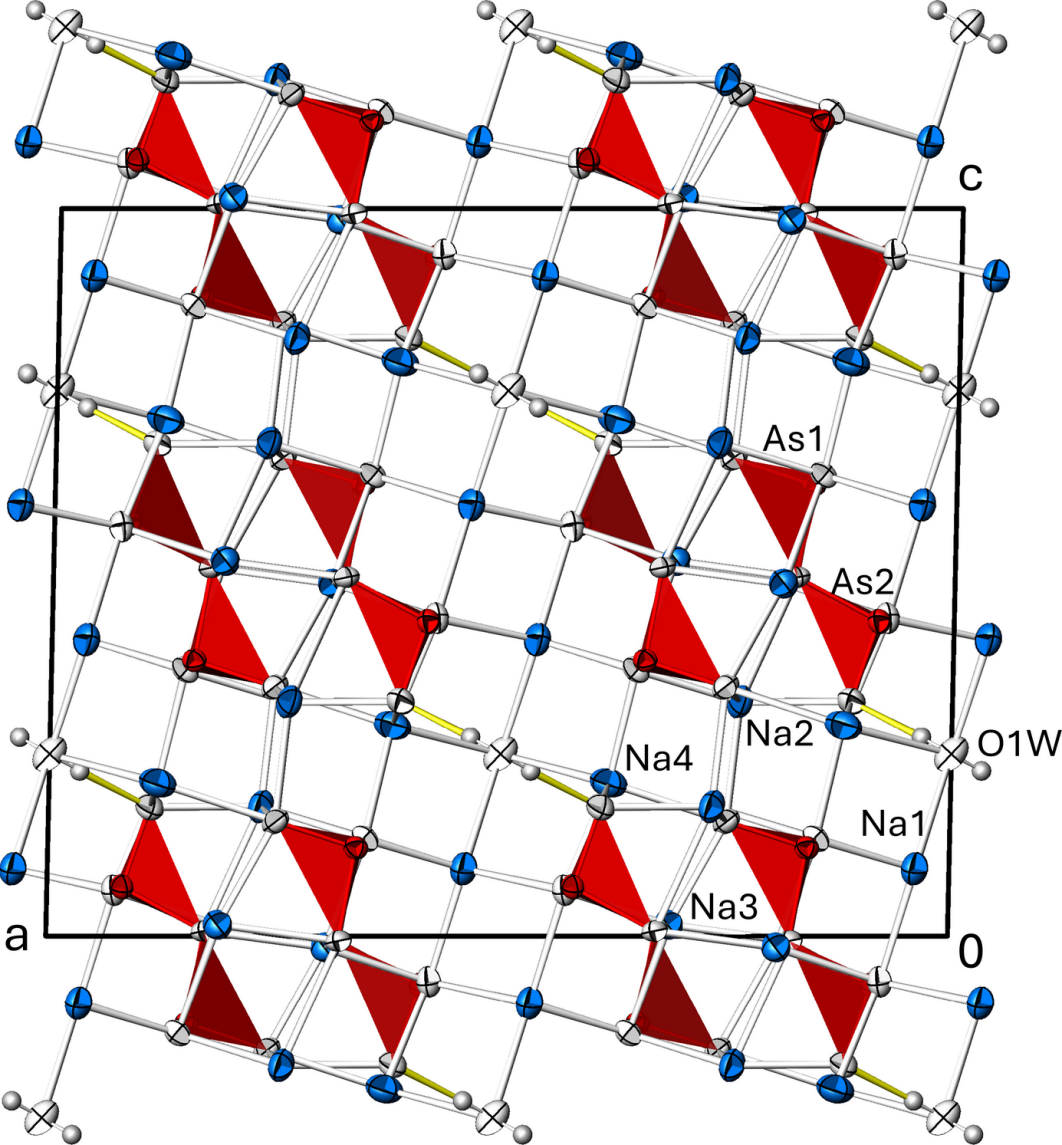
The crystal structure of Na_4_(As_2_O_5_)(H_2_O)_0.5_ in a projection along [0
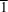
0]. The (As_2_O_5_)^4–^ anion is given in polyhedral representation; displacement ellipsoids are as in Fig. 1 ▸. Na—O bonds < 3.0 Å are displayed, and O—H⋯O hydrogen-bonding inter­actions are shown as yellow lines.

In the extended structure, the diarsenite groups are isolated from each other and arranged *via* inversion centres to form opposite pairs that are stacked along [010] (Fig. 3 ▸), whereby the anions are embedded in a framework of corner- and edge-sharing [NaO_4_] and [NaO_6_] polyhedra. The electron lone pairs of the As^III^ atoms are stereochemically active and point into the free space of the framework. The crystal structure of Na_4_(As_2_O_5_)(H_2_O)_0.5_ is consolidated by O—H⋯O hydrogen bonds between the water mol­ecule and a terminal O atom (O4) of the diarsenite anion as the acceptor atom (Table 3 ▸). Based on the *D*⋯*A* distance, the hydrogen-bonding inter­action is classified as of medium strength (Jeffrey, 1997 ▸).

To verify the validity of the structure model, bond-valence sums (BVS; Brown, 2002 ▸) were calculated using the *ECoN21* program (Ilinca, 2022 ▸). The BVS values (in valence units, v. u.) of the Na sites are listed in Table 1 ▸; those of the As and O atoms are As1 2.92, As2 3.01, O1 1.92, O2 1.84, O3 1.97, O4 1.61, O5 1.92, and O1*W* 0.50. The calculated values correspond to expectations [1.00 v. u. for Na, 3.00 v. u. for As, 2.00 v. u. for O] and also reflect the role of individual oxygen atoms in hydrogen-bonding inter­actions. Since the contributions of the H atoms to the bonding were not taken into account in the BVS calculations, the O1*W* atom of the water mol­ecule has a very low BVS value, and the O4 atom, which acts as the acceptor atom of the medium-strong hydrogen bond (Table 3 ▸), has a value significantly below 2.

## 3. Database survey

A search of the Inorganic Crystal Structure Database (ICSD; data release 2025-1; Zagorac *et al.*, 2019 ▸) for compounds in the system Na_2_O–As^III^_2_O_3_–H_2_O revealed five phases, *viz*. NaAsO_2_ (Menary, 1958 ▸; Lee & Harrison, 2004 ▸), Na_2_(H_2_As_4_O_8_), NaAsO_2_·4H_2_O, Na_2_(HAsO_3_)·5H_2_O and Na_5_(HAsO_3_)(AsO_3_)·12H_2_O (Sheldrick & Häusler, 1987 ▸). The first three phases consist of chains of polymetaarsenite anions, whereas the latter two phases contain discrete arsenite anions. Sodium compounds with pyroarsenite anions have not been reported to date, nor have those of other alkali metals. The title compound thus represents the first structurally characterized pyroarsenite of the alkali metals.

**Figure 4:**
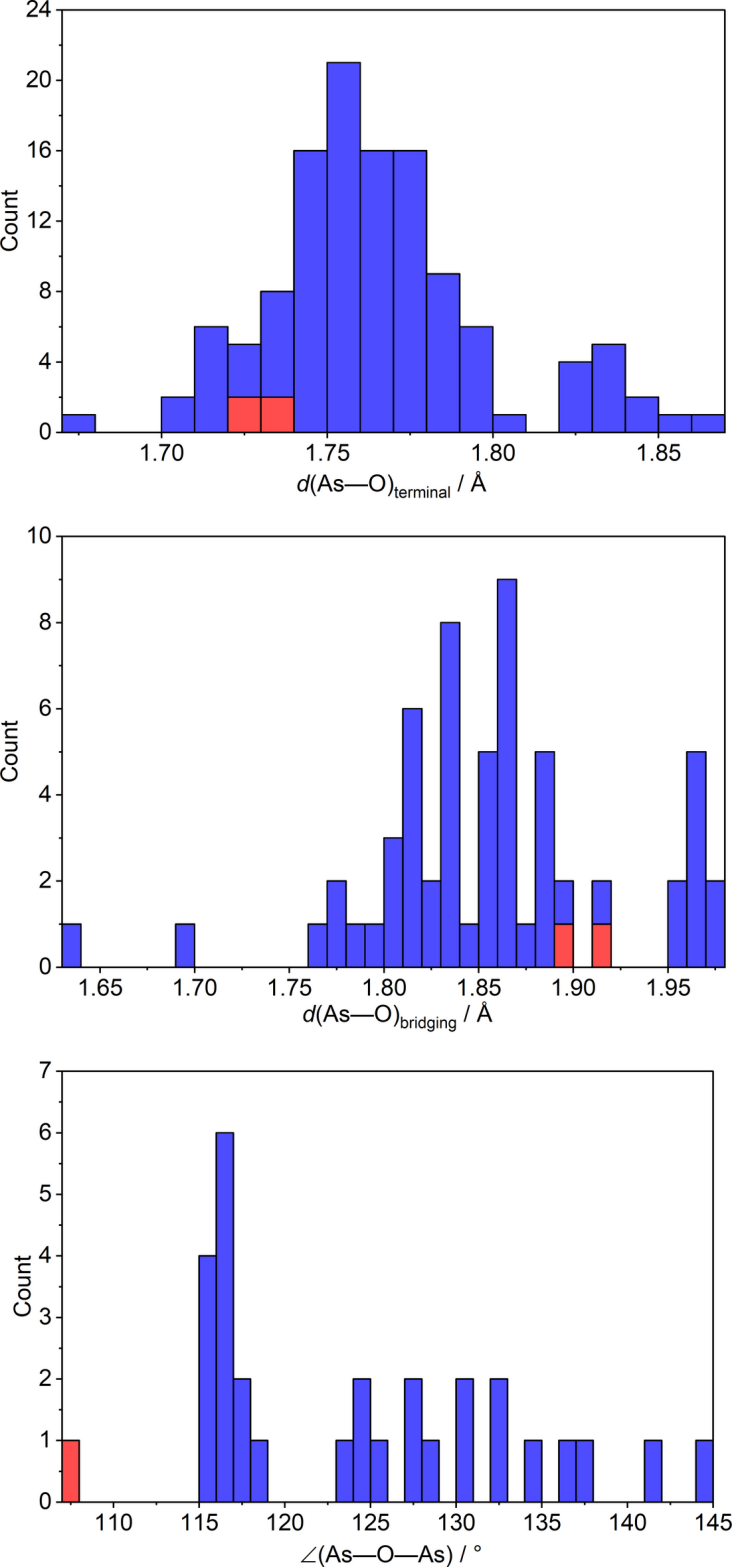
Histograms for the As—O_terminal_ and As—O_bridging_ bond lengths and As—O—As bridging angles in the crystal structures listed in Table 4 ▸; red colors refer to the title compound.

A further database search for inorganic compounds containing discrete pyroarsenite anions in their crystal structure yielded over 20 hits compiled in Table 4 ▸, including several minerals. Together with the title compound, this results in 30 individual (As_2_O_5_)^4–^ anions, whose averaged As—O bond lengths to terminal and bridging oxygen atoms and As—O—As bridging angles are listed. The structural features described in section 2 can also be observed for the vast majority of the other crystal structures comprising pyroarsenite anions, i.e., the presence of significantly longer As—O bonds to the bridging oxygen atoms compared to those involving terminal oxygen atoms. The mean As—O_terminal_ and As—O_bridging_ distances in all 30 pyroarsenite anions are 1.764 (33) Å and 1.856 (64) Å [overall mean As—O bond length 1.795 (63) Å]. However, the values of the As—O—As bridging angle in the listed pyroarsenite anions are highly variable [range 107.78 (13) to 144.12 (5)°], whereby Na_4_(As_2_O_5_)(H_2_O)_0.5_ has by far the smallest value of all structures. Histograms illustrating these features graphically can be found in Fig. 4 ▸.

## 4. Synthesis and crystallization

Na_8_(As_2_O_5_)_2_·H_2_O was first obtained serendipitously under hydro­flux conditions. Powders of Fe_2_O_3_ and As_2_O_3_ were mixed in a 1:2 ratio and combined with an excess of NaOH (98.5%_wt_) as a flux and a suitable amount of water to achieve a molar NaOH:H_2_O ratio of approximately 1:1. The reaction was carried out in a Teflon container placed in a steel autoclave that was heated to 483 K for 2 d. An off-white, highly water-soluble solid product was obtained, which also dissolved when placed in a methanol solution for a prolonged period of time. To remove the NaOH flux, the product was finally washed quickly in two stages, twice with dry methanol and twice with dry acetone. The shape of the obtained colourless crystals was rather unspecific, mostly plate- to block-like with rounded edges.

Na_4_(As_2_O_5_)·(H_2_O)_0.5_ was obtained specifically, i.e., without the addition of iron oxide, when As_2_O_3_ was heated directly with an excess of NaOH (approximate molar ratios As_2_O_3_: NaOH 1:12 and H_2_O: NaOH 1.5:1) in an autoclave under the same conditions.

## 5. Refinement

Crystal data, data collection and structure refinement details are summarized in Table 5 ▸. The position of the water H atom was clearly discernible from a difference-Fourier map. The O—H distance was restrained to 0.85 (1) Å, while the *U*_iso_(H) parameter was refined freely.

## Supplementary Material

Crystal structure: contains datablock(s) I. DOI: 10.1107/S2056989026002859/oo2017sup1.cif

Structure factors: contains datablock(s) I. DOI: 10.1107/S2056989026002859/oo2017Isup2.hkl

CCDC reference: 2538842

Additional supporting information:  crystallographic information; 3D view; checkCIF report

## Figures and Tables

**Table 1:** Selected geometric parameters (Å, °)

As1—O1	1.732 (3)	As2—O5	1.723 (3)
As1—O2	1.737 (3)	As2—O4	1.728 (3)
As1—O3	1.915 (3)	As2—O3	1.891 (3)
			
O1—As1—O2	102.68 (15)	O5—As2—O3	97.79 (16)
O1—As1—O3	98.42 (16)	O4—As2—O3	98.38 (12)
O2—As1—O3	97.34 (12)	As2—O3—As1	107.78 (13)
O5—As2—O4	102.92 (16)		

**Table 2:** Coordination environments (Å) around the Na^+^ cations in Na_4_(As_2_O_5_)(H_2_O)_0.5_

Atom	Coordination number	Polyhedron with idealized point group symmetry [in brackets] and deviation *δ* (in parentheses) from it	Range of Na—O bond lengths	Average Na—O bond length	Number of water mol­ecules in the first coordination sphere	Bond valence/valence units
Na1	4	heterodisphenoid [*mm*2] (4.159)	2.306 (4) – 2.559 (3)	2.408	1; O1*W*	0.76
Na2	6	didigonal scalenohedron [ 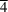 2*m*] (10.947)	2.259 (3) – 2.775 (3)	2.488	0; –	1.00
Na3	6	twisted trigonal prism [32] (3.999)	2.342 (3) – 2.613 (5)	2.464	0; –	0.98
Na4	6	monocapped trigonal anti­frustum [3*m*] (28.070)	2.409 (9) – 2.623 (4)	2.496	1; O1*W*	0.86

**Table 3:** Hydrogen-bond geometry (Å, °)

*D*—H⋯*A*	*D*—H	H⋯*A*	*D*⋯*A*	*D*—H⋯*A*
O1*W*—H1⋯O4	0.86 (1)	1.80 (1)	2.651 (4)	171 (6)

**Table 4:** As—O bond lengths (Å) and bridging angles (°) in the crystal structures of compounds with isolated pyroarsenite (As_2_O_5_)^4–^ groups

Compound (mineral name)	As—O_terminal_	As—O_bridging_	As—O—As	Reference
Na_4_(As_2_O_5_)·0.5H_2_O	1.732 (3), 1.737 (3), 1.723 (3), 1.728 (3)	1.915 (3), 1.891 (3)	107.78 (13)	This work
BaCo(As_2_O_5_)	2×1.716 (3), 2×1.736 (3)	1.837 (5), 1.809 (5)	130.9 (3)	David *et al.* (2014 ▸)
BaFe_2_(As_2_O_5_)(AsO_3_)(OH)	2×1.745 (13), 2×1.757 (12)	2×1.816 (7)	134.7 (10)	Leclercq *et al.* (2020 ▸)
Ba_2_Fe_2_O(As_2_O_5_)_2_	4×1.7503 (12)	2×1.8391 (11)	130.24 (14)	Leclercq *et al.* (2020 ▸)
Ba_2_(Ti^4+^V^3+^)(As_2_O_5_)_2_OF (bianchiniite)	4×1.7397 (15)	2×1.8377 (13)	127.12 (16)	Biagioni *et al.* (2021 ▸)
CaSb^5+^_2_(As_2_O_5_)_2_O_2_·10H_2_O (prachařite)	1.7633 (17), 1.7661 (16), 1.7611 (17), 1.7641 (17)	1.8079 (19), 1.8185 (18)	128.55 (9)	Kolitsch *et al.* (2023 ▸)
Fe^2+^Fe^3+^_3_(As_2_O_5_)_2_(AsO_3_) (schneiderhöhnite)	1.7680 (15), 1.7997 (16), 1.7936 (15), 1.7968 (15); 1.7689 (16), 1.7608 (15), 1.7485 (15), 1.7844 (15)	1.7926 (16), 1.7648 (15); 1.8610 (15), 1.8075 (16)	132.9 (2), 136.8 (2)	Cooper & Hawthorne (2016 ▸)
Fe^3+^_3_(AsO_2_)_4_(As_2_O_5_)(OH) (karibibite)	2×1.77 (2), 2×1.79 (2)	2×1.77 (2)	141 (3)	Colombo *et al.* (2017 ▸)
Fe_3_(As_2_O_5_)(AsO_3_)Cl	1.779 (7), 1.772 (8), 1.825 (7), 1.766 (7)	1.834 (7), 1.811 (9)	125.2 (4)	Leclercq *et al.* (2020 ▸)
In_2_(As_2_O_5_)Cl_2_	1.756 (5), 1.804 (8), 1.742 (6), 1.786 (7)	1.912 (7), 1.827 (6)	124.9 (5)	Jiang *et al.* (2011 ▸)
In_4_(As_2_O_5_)(As_3_O_7_)Br_3_	1.734 (9), 1.782 (8), 1.777 (10), 1.780 (8)	1.881 (9), 1.814 (8)	124.3 (5)	Jiang *et al.* (2011 ▸)
Mn_2_(As_2_O_5_)	1.727 (4), 1.736 (4), 1.740 (3), 1.752 (4), 1.709 (4), 1.763 (3), 1.722 (4), 1.779 (3)	1.872 (4), 1.838 (4), 1.860 (4), 1.834 (4)	116.04 (16), 137.33 (19)	Priestner *et al.* (2019 ▸)
Nd_4_(A_2_O_5_)_2_(As_4_O_8_)	1.716 (3), 1.778 (4), 1.719 (4), 1.783 (4)	1.861 (4), 1.880 (4)	118.2 (2)	Ben Hamida *et al.* (2005 ▸)
[(Mo^6+^O_2_)_2_(H_2_O)_2_(As_2_O_5_]·3H_2_O (vajdakite)	1.750 (6), 1.822 (6), 1.778 (6), 1.793 (6)	1.786 (5), 1.817 (5)	127.6 (3)	Ondruš *et al.* (2002 ▸)
Pb_2_As_2_O_5_ (paulmooreite)	1.747 (9), 1.750 (9), 1.733 (9), 1.772 (8)	1.826 (9), 1.842 (9)	123.0 (5)	Araki *et al.* (1980 ▸)
Pb_8_OCl_6_(As_2_O_5_)_2_ (gebhardite)	1.762 (2), 1.823 (2), 1.674 (2), 1.792 (2); 1.757 (2), 1.757 (2), 1.756 (2), 1.866 (2)	1.888 (2), 1.6323 (19); 1.693 (2), 1.890 (2)	132.85 (6); 144.12 (5)	Klaska & Gebert (1982 ▸)
*RE*_3_Cl_2_(AsO_3_)(As_2_O_5_) *RE* = Eu; Gd	1.776 (5), 1.827 (5), 1.751 (5), 1.753 (5); 1.772 (2), 1.831 (2), 1.741 (2); 1.749 (2)	1.864 (5), 1.968 (5); 1.855 (2), 1.972 (2)	116.0 (3), 115.97 (13)	Schander *et al.* (2024 ▸)
*RE*_3_Br_2_(AsO_3_)(As_2_O_5_) *RE* = Y, Dy–Yb	1.736 (10) – 1.858 (9)	1.858 (9) – 1.971 (7)	115.2 (5) – 116.0 (5)	Locke *et al.* (2025 ▸).
Sm_3_Cl_2_(As_2_O_5_)(AsO_3_)	1.769 (7), 1.833 (7), 1.753 (6), 1.765 (7)	1.866 (6), 1.969 (6)	116.2 (3)	Goerigk *et al.* (2020 ▸)
Sm_4_(A_2_O_5_)_2_(As_4_O_8_)	1.719 (2), 1.787 (2), 1.714 (2), 1.774 (3)	1.883 (2), 1.862 (3)	117.7 (2)	Kang & Schleid (2006 ▸)
Sm_4_(A_2_O_5_)_2_(As_4_O_8_)	1.709 (7), 1.778 (8), 1.721 (8), 1.783 (8)	1.850 (8), 1.886 (8)	117.7 (4)	Ben Hamida *et al.* (2005 ▸)

**Table 5:** Experimental details

Crystal data	
Chemical formula	[Na_4_(As_2_O_5_)(H_2_O)_0.5_]
*M*_r_	330.81
Crystal system, space group	Monoclinic, *C*2/*c*
Temperature (K)	301
*a*, *b*, *c* (Å)	18.283 (3), 5.0747 (9), 14.740 (3)
β (°)	91.256 (6)
*V* (Å^3^)	1367.3 (4)
*Z*	8
Radiation type	Mo *K*α
μ (mm^−1^)	10.00
Crystal size (mm)	0.04 × 0.03 × 0.02

Data collection	
Diffractometer	Bruker APEXII CCD
Absorption correction	Multi-scan (*SADABS*; Krause *et al.*, 2015 ▸)
*T*_min_, *T*_max_	0.660, 0.746
No. of measured, independent and observed [*I* > 2σ(*I*)] reflections	10323, 1910, 1193
*R*_int_	0.069
(sin θ/λ)_max_ (Å^−1^)	0.695

Refinement	
*R*[*F*^2^ > 2σ(*F*^2^)], *wR*(*F*^2^), *S*	0.033, 0.058, 1.02
No. of reflections	1910
No. of parameters	109
No. of restraints	1
H-atom treatment	All H-atom parameters refined
Δρ_max_, Δρ_min_ (e Å^−3^)	0.72, −0.79

Computer programs: *APEX4* and *SAINT* (Bruker, 2022 ▸), *SHELXT* (Sheldrick, 2015*a* ▸), *SHELXL* (Sheldrick, 2015*b* ▸), *ATOMS for Windows* (Dowty, 2006 ▸) and *publCIF* (Westrip, 2010 ▸).

## supplementary crystallographic information

### Tetrasodium pyroarsenite hemihydrate Crystal data

**Table:**

[Na_4_(As_2_O_5_)(H_2_O)_0.5_]	*F*(000) = 1240
*M**_r_* = 330.81	*D*_x_ = 3.214 Mg m^−^^3^
Monoclinic, *C*2/*c*	Mo *K*α radiation, λ = 0.71073 Å
*a* = 18.283 (3) Å	Cell parameters from 1379 reflections
*b* = 5.0747 (9) Å	θ = 2.2–28.9°
*c* = 14.740 (3) Å	µ = 10.00 mm^−^^1^
β = 91.256 (6)°	*T* = 301 K
*V* = 1367.3 (4) Å^3^	Block, colourless
*Z* = 8	0.04 × 0.03 × 0.02 mm

### Tetrasodium pyroarsenite hemihydrate Data collection

**Table:**

Bruker APEXII CCD diffractometer	1193 reflections with *I* > 2σ(*I*)
ω– and φ–scans	*R*_int_ = 0.069
Absorption correction: multi-scan (*SADABS*; Krause *et al.*, 2015)	θ_max_ = 29.6°, θ_min_ = 2.2°
*T*_min_ = 0.660, *T*_max_ = 0.746	*h* = −25→25
10323 measured reflections	*k* = −7→7
1910 independent reflections	*l* = −20→20

### Tetrasodium pyroarsenite hemihydrate Refinement

**Table:**

Refinement on *F*^2^	1 restraint
Least-squares matrix: full	Hydrogen site location: difference Fourier map
*R*[*F*^2^ > 2σ(*F*^2^)] = 0.033	All H-atom parameters refined
*wR*(*F*^2^) = 0.058	*w* = 1/[σ^2^(*F*_o_^2^) + 4.6824*P*] where *P* = (*F*_o_^2^ + 2*F*_c_^2^)/3
*S* = 1.02	(Δ/σ)_max_ < 0.001
1910 reflections	Δρ_max_ = 0.72 e Å^−^^3^
109 parameters	Δρ_min_ = −0.79 e Å^−^^3^

### Tetrasodium pyroarsenite hemihydrate Special details

**Table:**

Geometry. All esds (except the esd in the dihedral angle between two l.s. planes) are estimated using the full covariance matrix. The cell esds are taken into account individually in the estimation of esds in distances, angles and torsion angles; correlations between esds in cell parameters are only used when they are defined by crystal symmetry. An approximate (isotropic) treatment of cell esds is used for estimating esds involving l.s. planes.

### Tetrasodium pyroarsenite hemihydrate Fractional atomic coordinates and isotropic or equivalent isotropic displacement parameters (Å^2^)

**Table:**

	*x*	*y*	*z*	*U*_iso_*/*U*_eq_	
Na1	0.03814 (10)	0.4524 (4)	0.09242 (14)	0.0176 (4)	
Na2	0.23554 (8)	0.0157 (5)	0.32203 (11)	0.0163 (4)	
Na3	0.30984 (9)	0.0008 (5)	0.01553 (11)	0.0149 (4)	
Na4	0.37938 (11)	0.0460 (4)	0.21031 (13)	0.0207 (5)	
As1	0.15826 (2)	0.01960 (12)	0.62085 (3)	0.00966 (12)	
As2	0.08337 (2)	−0.01329 (12)	0.43240 (3)	0.01006 (11)	
O1	0.14842 (16)	0.3569 (6)	0.6337 (2)	0.0111 (8)	
O2	0.24805 (15)	−0.0336 (7)	0.65721 (19)	0.0132 (7)	
O3	0.17524 (14)	0.0122 (8)	0.49315 (17)	0.0133 (6)	
O4	0.11217 (15)	0.0392 (7)	0.3230 (2)	0.0157 (7)	
O5	0.07415 (16)	−0.3510 (7)	0.4372 (2)	0.0132 (8)	
O1W	0.000000	0.3006 (10)	0.250000	0.0189 (12)	
H1	0.034 (2)	0.202 (9)	0.273 (4)	0.044 (19)*	

### Tetrasodium pyroarsenite hemihydrate Atomic displacement parameters (Å^2^)

**Table:**

	*U*^11^	*U*^22^	*U*^33^	*U*^12^	*U*^13^	*U*^23^
Na1	0.0124 (8)	0.0218 (10)	0.0184 (9)	0.0026 (10)	−0.0012 (7)	−0.0004 (10)
Na2	0.0115 (8)	0.0186 (10)	0.0186 (9)	−0.0027 (11)	−0.0024 (7)	0.0006 (11)
Na3	0.0146 (8)	0.0152 (9)	0.0149 (9)	−0.0013 (11)	0.0007 (7)	−0.0017 (11)
Na4	0.0240 (9)	0.0239 (12)	0.0143 (10)	0.0033 (11)	0.0035 (8)	−0.0003 (10)
As1	0.0102 (2)	0.0097 (3)	0.0091 (2)	−0.0005 (2)	0.00026 (17)	−0.0004 (3)
As2	0.0089 (2)	0.0107 (2)	0.0106 (2)	0.0009 (2)	0.00064 (17)	0.0001 (2)
O1	0.0125 (18)	0.0092 (18)	0.0118 (18)	0.0013 (12)	0.0030 (14)	−0.0007 (13)
O2	0.0124 (14)	0.0161 (17)	0.0109 (14)	0.0038 (15)	−0.0026 (11)	−0.0028 (15)
O3	0.0119 (14)	0.0204 (17)	0.0076 (13)	−0.0027 (18)	−0.0007 (11)	0.0005 (17)
O4	0.0134 (15)	0.0215 (19)	0.0122 (15)	0.0007 (15)	0.0018 (12)	0.0038 (15)
O5	0.0101 (19)	0.013 (2)	0.016 (2)	−0.0009 (13)	0.0010 (16)	−0.0006 (13)
O1W	0.019 (3)	0.017 (3)	0.020 (3)	0.000	−0.007 (2)	0.000

### Tetrasodium pyroarsenite hemihydrate Geometric parameters (Å, º)

**Table:**

Na1—O1^i^	2.306 (4)	Na3—O3^vi^	2.613 (5)
Na1—O5^ii^	2.316 (4)	Na4—O5^vi^	2.409 (4)
Na1—O5^iii^	2.450 (4)	Na4—O1^iv^	2.416 (4)
Na1—O1W	2.559 (3)	Na4—O2^iii^	2.510 (3)
Na2—O4	2.259 (3)	Na4—O4^vi^	2.556 (4)
Na2—O1^iv^	2.299 (3)	Na4—O1W^viii^	2.588 (3)
Na2—O2^iii^	2.447 (3)	Na4—O4^vii^	2.623 (4)
Na2—O2^v^	2.483 (4)	As1—O1	1.732 (3)
Na2—O2^iv^	2.662 (4)	As1—O2	1.737 (3)
Na2—O3	2.775 (3)	As1—O3	1.915 (3)
Na3—O5^vi^	2.342 (3)	As2—O5	1.723 (3)
Na3—O2^iii^	2.402 (3)	As2—O4	1.728 (3)
Na3—O1^vii^	2.455 (4)	As2—O3	1.891 (3)
Na3—O3^iii^	2.477 (3)	O1W—H1	0.860 (10)
Na3—O3^vii^	2.498 (5)	O1W—H1^ix^	0.860 (10)
			
O1^i^—Na1—O5^ii^	129.43 (13)	Na2^iv^—O1—Na3^vi^	82.39 (11)
O1^i^—Na1—O5^iii^	94.78 (12)	Na1^x^—O1—Na3^vi^	85.74 (12)
O5^ii^—Na1—O5^iii^	99.78 (12)	Na4^iv^—O1—Na3^vi^	150.20 (15)
O1^i^—Na1—O1W	98.11 (11)	As1—O2—Na3^xi^	100.39 (13)
O5^ii^—Na1—O1W	92.44 (11)	As1—O2—Na2^xi^	101.09 (13)
O5^iii^—Na1—O1W	150.34 (15)	Na3^xi^—O2—Na2^xi^	156.45 (14)
O4—Na2—O1^iv^	153.99 (15)	As1—O2—Na2^v^	107.58 (15)
O4—Na2—O2^iii^	96.86 (11)	Na3^xi^—O2—Na2^v^	96.75 (13)
O1^iv^—Na2—O2^iii^	99.53 (12)	Na2^xi^—O2—Na2^v^	85.87 (12)
O4—Na2—O2^v^	99.75 (13)	As1—O2—Na4^xi^	172.49 (19)
O1^iv^—Na2—O2^v^	97.70 (12)	Na3^xi^—O2—Na4^xi^	78.92 (10)
O2^iii^—Na2—O2^v^	98.35 (13)	Na2^xi^—O2—Na4^xi^	78.52 (10)
O4—Na2—O2^iv^	93.27 (13)	Na2^v^—O2—Na4^xi^	79.91 (10)
O1^iv^—Na2—O2^iv^	65.71 (11)	As1—O2—Na2^iv^	89.16 (13)
O2^iii^—Na2—O2^iv^	93.71 (13)	Na3^xi^—O2—Na2^iv^	88.71 (12)
O2^v^—Na2—O2^iv^	161.01 (14)	Na2^xi^—O2—Na2^iv^	82.07 (11)
O4—Na2—O3	65.04 (10)	Na2^v^—O2—Na2^iv^	161.02 (14)
O1^iv^—Na2—O3	97.26 (11)	Na4^xi^—O2—Na2^iv^	83.35 (11)
O2^iii^—Na2—O3	161.88 (10)	As2—O3—As1	107.78 (13)
O2^v^—Na2—O3	86.05 (12)	As2—O3—Na3^xi^	158.62 (15)
O2^iv^—Na2—O3	87.01 (12)	As1—O3—Na3^xi^	92.96 (10)
O5^vi^—Na3—O2^iii^	99.15 (12)	As2—O3—Na3^vi^	98.08 (15)
O5^vi^—Na3—O1^vii^	93.73 (11)	As1—O3—Na3^vi^	92.93 (14)
O2^iii^—Na3—O1^vii^	163.93 (14)	Na3^xi^—O3—Na3^vi^	85.65 (12)
O5^vi^—Na3—O3^iii^	159.87 (15)	As2—O3—Na3^vii^	90.08 (14)
O2^iii^—Na3—O3^iii^	68.45 (10)	As1—O3—Na3^vii^	94.99 (14)
O1^vii^—Na3—O3^iii^	101.55 (11)	Na3^xi^—O3—Na3^vii^	82.85 (12)
O5^vi^—Na3—O3^vii^	103.59 (12)	Na3^vi^—O3—Na3^vii^	166.34 (14)
O2^iii^—Na3—O3^vii^	99.63 (12)	As2—O3—Na2	86.35 (10)
O1^vii^—Na3—O3^vii^	67.81 (10)	As1—O3—Na2	165.85 (13)
O3^iii^—Na3—O3^vii^	94.35 (12)	Na3^xi^—O3—Na2	73.01 (8)
O5^vi^—Na3—O3^vi^	66.47 (11)	Na3^vi^—O3—Na2	84.33 (11)
O2^iii^—Na3—O3^vi^	91.46 (12)	Na3^vii^—O3—Na2	85.28 (11)
O1^vii^—Na3—O3^vi^	102.52 (11)	As2—O4—Na2	108.81 (14)
O3^iii^—Na3—O3^vi^	97.15 (12)	As2—O4—Na4^vii^	92.81 (15)
O3^vii^—Na3—O3^vi^	166.34 (14)	Na2—O4—Na4^vii^	83.25 (12)
O5^vi^—Na4—O1^iv^	154.17 (13)	As2—O4—Na4^vi^	110.26 (16)
O5^vi^—Na4—O2^iii^	94.47 (11)	Na2—O4—Na4^vi^	89.30 (13)
O1^iv^—Na4—O2^iii^	94.71 (12)	Na4^vii^—O4—Na4^vi^	156.92 (15)
O5^vi^—Na4—O4^vi^	65.80 (12)	As2—O5—Na1^xii^	120.53 (16)
O1^iv^—Na4—O4^vi^	89.87 (12)	As2—O5—Na3^vii^	104.04 (15)
O2^iii^—Na4—O4^vi^	91.49 (12)	Na1^xii^—O5—Na3^vii^	135.32 (16)
O5^vi^—Na4—O1W^viii^	89.59 (10)	As2—O5—Na4^vii^	98.18 (15)
O1^iv^—Na4—O1W^viii^	94.54 (10)	Na1^xii^—O5—Na4^vii^	93.74 (13)
O2^iii^—Na4—O1W^viii^	149.65 (16)	Na3^vii^—O5—Na4^vii^	82.18 (11)
O4^vi^—Na4—O1W^viii^	117.37 (14)	As2—O5—Na1^xi^	105.99 (15)
O5^vi^—Na4—O4^vii^	91.18 (12)	Na1^xii^—O5—Na1^xi^	80.22 (12)
O1^iv^—Na4—O4^vii^	113.12 (13)	Na3^vii^—O5—Na1^xi^	85.10 (11)
O2^iii^—Na4—O4^vii^	88.68 (11)	Na4^vii^—O5—Na1^xi^	154.79 (16)
O4^vi^—Na4—O4^vii^	156.92 (15)	Na1^ix^—O1W—Na1	144.9 (2)
O1W^viii^—Na4—O4^vii^	61.14 (12)	Na1^ix^—O1W—Na4^vi^	84.13 (10)
O1—As1—O2	102.68 (15)	Na1—O1W—Na4^vi^	79.19 (10)
O1—As1—O3	98.42 (16)	Na1^ix^—O1W—Na4^xiii^	79.19 (10)
O2—As1—O3	97.34 (12)	Na1—O1W—Na4^xiii^	84.13 (10)
O5—As2—O4	102.92 (16)	Na4^vi^—O1W—Na4^xiii^	122.5 (2)
O5—As2—O3	97.79 (16)	Na1^ix^—O1W—H1	91 (4)
O4—As2—O3	98.38 (12)	Na1—O1W—H1	109 (4)
As1—O1—Na2^iv^	102.22 (15)	Na4^vi^—O1W—H1	66 (4)
As1—O1—Na1^x^	118.57 (16)	Na4^xiii^—O1W—H1	166 (4)
Na2^iv^—O1—Na1^x^	138.84 (16)	Na1^ix^—O1W—H1^ix^	109 (4)
As1—O1—Na4^iv^	109.29 (15)	Na1—O1W—H1^ix^	91 (4)
Na2^iv^—O1—Na4^iv^	83.37 (12)	Na4^vi^—O1W—H1^ix^	166 (4)
Na1^x^—O1—Na4^iv^	87.96 (12)	Na4^xiii^—O1W—H1^ix^	66 (4)
As1—O1—Na3^vi^	99.26 (14)	H1—O1W—H1^ix^	109 (8)

Symmetry codes: (i) *x*, −*y*+1, *z*−1/2; (ii) −*x*, *y*+1, −*z*+1/2; (iii) *x*, −*y*, *z*−1/2; (iv) −*x*+1/2, −*y*+1/2, −*z*+1; (v) −*x*+1/2, −*y*−1/2, −*z*+1; (vi) −*x*+1/2, *y*+1/2, −*z*+1/2; (vii) −*x*+1/2, *y*−1/2, −*z*+1/2; (viii) *x*+1/2, *y*−1/2, *z*; (ix) −*x*, *y*, −*z*+1/2; (x) *x*, −*y*+1, *z*+1/2; (xi) *x*, −*y*, *z*+1/2; (xii) −*x*, *y*−1, −*z*+1/2; (xiii) *x*−1/2, *y*+1/2, *z*.

### Tetrasodium pyroarsenite hemihydrate Hydrogen-bond geometry (Å, º)

**Table:**

*D*—H···*A*	*D*—H	H···*A*	*D*···*A*	*D*—H···*A*
O1*W*—H1···O4	0.86 (1)	1.80 (1)	2.651 (4)	171 (6)
